# Numerical investigation of the mechano-electro-chemical effect of X100 buried pipelines with pre-existing corrosion defects

**DOI:** 10.1016/j.heliyon.2023.e22440

**Published:** 2023-11-20

**Authors:** Ghadeer Mubarak, Ibrahim Gadala, Imad Barsoum, Akram AlFantazi

**Affiliations:** a Department of Chemical Engineering, Khalifa University, Abu Dhabi, 12788, United Arab Emirates; b Department of Mechanical Engineering, Khalifa University, Abu Dhabi, 12788, United Arab Emirates; c Center for Applied Research, NOVA Chemicals Corporation, 2928 16 St NE, Calgary, AB T2E 7K7, Canada; d Advanced Digital & Additive Manufacturing Center, Khalifa University, Abu Dhabi, 12788, United Arab Emirates

**Keywords:** Mechano-electrochemical interactions, X100, Corrosion growth rate, Anodic current density, Crack propagation, Extended finite element analysis

## Abstract

This study investigates the corrosion kinetics and crack propagation in buried transmission pipelines made of high-strength low alloy steel API X100. Despite its cost-effectiveness and ability to withstand high operating conditions without increasing pipe wall thickness, the corrosion kinetics in near-neutral pH environments for this steel grade is not fully understood. To address this gap, two numerical models were developed. The first model, using COMSOL Multiphysics v5.6, showed higher electrolyte potential at the corrosion defect center due to stress-induced defect growth, increasing corrosion susceptibility. The second model, employing the XFEM approach, evaluated crack initiation, propagation, and von Mises stress distribution along the crack path. This research contributes to a better understanding of corrosion and crack behavior in corroded pipelines, aiding in their performance improvement in near-neutral pH soil environments.

## Highlights

• Investigation of X100 steel corrosion in near-neutral pH.
• Mechanisms of stress-induced corrosion susceptibility.
• Analysis of crack initiation, propagation, and stress distribution.
• Insights for enhancing pipeline performance in such environments.

## 1 Introduction

Transmission pipelines, both onshore and offshore, are widely utilized for the economical transportation of oil and gas, driven by increasing global consumption. However, these pipelines are prone to internal and external corrosion, posing significant risks to their structural integrity and operational safety [[1], [2], [3], [4], [5], [6]]. Internal corrosion occurs due to impurities present in crude oil and natural gas, such as highly corrosive carbon dioxide, hydrogen sulfide, and water. Conversely, external corrosion predominantly affects buried pipelines, influenced by corrosive soil environments [[7], [8], [9]]. Consequently, material degradation in pipelines can lead to a loss of mechanical properties, including strength, ductility, and impact strength. Over time, this can result in material loss, thickness reduction, and ultimately, pipeline failure. Therefore, it is crucial to assess the remaining strength of pipelines containing corrosion defects. Pipelines experience complex stress and strain conditions. Internal fluid pressure generates circumferential stress (hoop stress) within the pipeline, while the external environment induces longitudinal stress. However, when the pipeline contains corrosion or mechanical defects, the stress and strain distribution differs.

The combination of seawater, oxygen, and various contaminants present in marine environments can accelerate the corrosion process as well. Previous research on the E690 high-strength steel lattice corrugated panel under uniform corrosion in a marine environment reveals that corrosion on the inner surface significantly reduces its mechanical properties, while corrosion on the outer surface has minimal effect [10]. Longitudinal loading exhibits higher bending stiffness and peak load compared to transverse loading, with both decreasing as corrosion progresses. Building upon these findings, Liu [11] aimed to expand their understanding of corrosion-induced degradation in the E690 panel, specifically investigating the influence of pitting corrosion. It was found that pitting corrosion of the E690 panel core significantly reduces its quasi-static compression mechanical properties, while corrosion pits on the outer panel have no evident effect.

Several studies have focused on mechano-electrochemical investigations on pipelines with defects, aiming to understand the relationship between mechanical stress/strain conditions and their impact on corrosion in corroded pipelines. In the case of corrosion defects in X100 pipelines, Gao et al. [3] conducted an experimental and numerical study to analyze the mechano-electrochemical effects. The study revealed that an increase in longitudinal strain resulted in an overall stress increase across the pipe wall. However, an increase in corrosion defect depth led to localized stress concentration at the center of the defect, while stress concentration on the sides of the defect decreased with greater defect depth. Furthermore, the study demonstrated that an increase in corrosion defect depth and tensile strain contributed to higher local corrosion activity in the pipeline. This behavior can be attributed to the presence of local galvanic cells, where regions with higher stress concentrations (defect center) act as anodes, and regions with lower stress concentrations act as cathodes.

In reality, corrosion defects occur in clusters, which exacerbates the situation compared to dealing with isolated or adjacent defects that can interact and reduce the pipeline's failure pressure [12]. Kiu et al. [4] investigated the mechano-electrochemical interaction of circumferentially aligned corrosion defects on the pipeline. Finite element modeling was employed, and the results demonstrated that this interaction intensified at the corrosion defect center and inside edges, resulting in the maximum von Mises stress, the most negative corrosion potential, and the highest anodic current density. These factors collectively enhanced the local corrosion activity of the pipeline. This study highlights a significant finding regarding the accelerated corrosion of corrosion defects when they interact with specific geometrical orientations, compared to individual defects. The validation of this finding was based on comparing defect growth over time using periodic in-line inspections. Additionally, the same research group conducted another study on multiple longitudinally aligned corrosion defects [13], which examines how adjacent corrosion defects on pipelines interact with each other, specifically focusing on the impact of the length and spacing of the defects on their mechanical-electrochemical (M-E) interaction. The findings demonstrate that as the length of a corrosion defect increases, while keeping its depth constant, raises the local stress, shifts the corrosion potential in a negative direction, and increases the anodic current density both at the defect center and in the area between the adjacent defects. It has been observed that the applied mechanical stress can enhance the electrochemical activity of steels, causing the equilibrium potential to shift negatively. With increasing defect length, the corrosion potential becomes more negative due to higher level of stress concentration. The presence of corrosion defects on the steel creates localized galvanic cells, where the defect center acts as the anode and the defect boundary serves as the cathode. The anodic dissolution rate at the defect center is amplified by the high stress/strain concentration. Additionally, the spacing between adjacent corrosion defects is a critical factor influencing their M-E interaction. Under axial loading, which involves a uniaxial stress state, the stress between two neighboring defects is minimal. However, when the pipeline experiences hoop stress in the circumferential direction, the displacement of corrosion defects is greater than that of the uncorroded region, resulting in additional bending moments and significantly increased local stress at the defects and the area between them. Consequently, the distribution of corrosion potential and anodic current density differs depending on whether axial or hoop stress is present. The study suggests that determining the critical spacing between interacting corrosion defects should not solely rely on stress field calculations but also consider the electric field generated by the M-E effect.

Understanding corrosion in X100 pipeline steel within near-neutral pH soil environments and under stress conditions is crucial for ensuring the safety, reliability, cost-effectiveness, and environmental sustainability of pipelines. It enables the development of appropriate corrosion control strategies, optimization of pipeline design, and compliance with regulatory standards. Hence, the principal aim of this work is to contribute towards an extensive comprehension and modeling of corrosion mechanisms, as well as crack propagation phenomena, in buried X100 pipelines operating within near-neutral soil environments.

## 2 Methodology

### 2.1 Electrochemical model

**Fig. 1:**
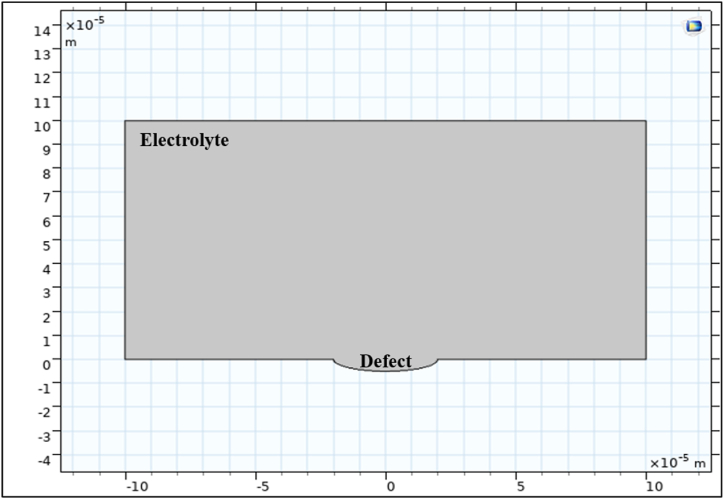
2D representation of the electrolyte-corrosion defect system.

The electrochemical finite element model (FEM) implemented in this study utilizes COMSOL® Multiphysics version 5.4a software. The model employed is time-dependent, accounting for the deformed geometry and Secondary Current Distribution Multiphysics. Fig. 1 illustrates the two-dimensional (2D) representation of the electrolyte system, displaying the presence of a pre-existing corrosion defect on the external surface of the pipeline.

**Fig. 2:**
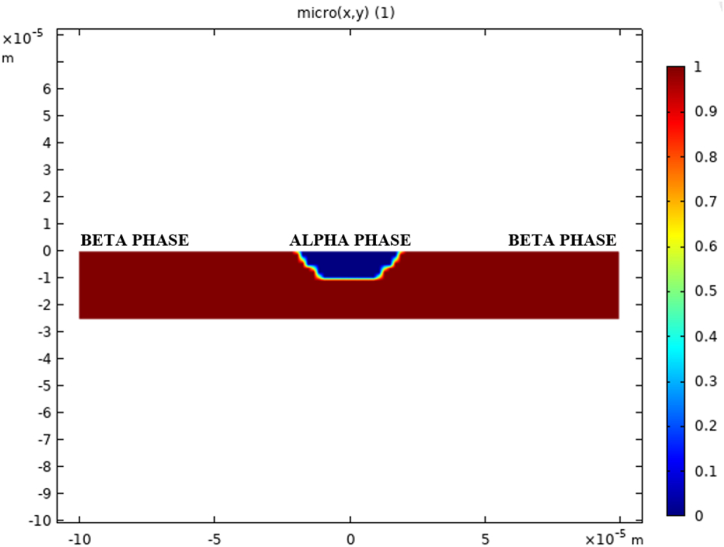
Representation of the interpolation function 'micro'.

In the FEM model, the defect is defined as 'micro' and is integrated over the electrode surface using an interpolation function adapted from Ref. [14]. The interpolated function 'micro' (Fig. 2) comprises the alpha and beta phases exposed to the electrolyte solution. The model assumes that the anodic dissolution reaction occurs at the alpha phase, while the water reduction for near-neutral pH conditions takes place at the beta phase surface, following reactions (1) and (2) as shown below [15]:

(R.1) $$\text{Fe}\to {\text{Fe}}^{2+}+2e^{-}$$

(R.2) $$2H^{+}+2e^{-}\to H_{2}$$

Consequently, material loss occurs only at the alpha phase, which represents the dissolving phase, while the beta phase surface remains structurally intact. However, electrochemically, the beta phase is protected through a small-scale galvanic cell.

To solve the electrolyte potential within the electrolyte domain, the Secondary Current Distribution Multiphysics interface is employed, based on equations (R.1), (R.2) [16]:

(Eq.1) $$i_{l}=-\sigma_{l}\nabla \varphi_{l}$$

(Eq.1) $$\nabla ∙\;i_{l}=0$$

Here $i_{l}$ (A/m^2^) represents the electrolyte current density as a vector, and $\sigma_{l}$ (S/m) is the assumed constant electrolyte conductivity of 0.096 S/m [17]. The defect surface boundary is considered the electrode surface, while the default insulation condition is applied to all other boundaries, satisfying the condition $n∙i_{l}=0$, where *n* is the normal vector of the domain. An electrode surface boundary node is assigned with an added dissolving depositing species at the electrode surface. This establishes the boundary condition for the electrolyte potential as follows [16]:

$$n∙i_{l}=\sum_{m}i_{loc,m}+i_{dl}$$

where $i_{loc,m}$ (A/m^2^) denotes the local electrode reaction current density. The dissolution at the electrode surface, occurring only in the normal direction, is evaluated using the following equation [16]:

$$n∙\;\frac{\partial x}{\partial t}=\sum_{i}\frac{R_{dep,i,m}M_{i}}{\rho_{i}}$$

where $M_{i}$ represents the molar mass of steel (55.845 kg/mol), $\rho_{i}$ denotes the density of steel (7750 kg/m^3^) and $R_{\text{dep},i,m}$ is evaluated using the following equation:

$$R_{dep,i,m}=-\frac{\upsilon_{dep,i,m}i_{loc,m}}{n_{m}F}$$

Here, $\upsilon_{dep,i,m}$ represents the stoichiometric coefficient, and $n_{m}$ is the number of electrons participating in the electrode reaction. The electrode kinetics for modeling the reactions at the alpha and beta phases on the electrode surfaces are expressed as follows:

$$i_{alpha}=f\left(\varphi_{s,ext}-\varphi_{1})\times (1-\text{micro}\left(x,y\right)\right)$$

$$i_{beta}=f\left(\varphi_{s,ext}-\varphi_{1}\right)\times \left(\text{micro}\left(x,y\right)\right)$$

The level set function (1−micro(x,y)) ensures that the local anodic current density is applied solely. Similarly, the inclusion of the expression micro(x,y) in the beta phase electrode kinetics expression ensures that the local cathodic current density is solely applied at the beta phase of the electrode surface. To establish the relationship between the local current density and the electrolyte potential for the alpha and beta phases at the electrode surface, a piecewise cubic interpolation function is incorporated into the model. This interpolation function allows for the determination of the current density based on the electrolyte potential values. The incorporation of the experimental electrochemical polarization data derived from polarization tests conducted on stressed X70 steel samples [18] has a profound impact on our model. By utilizing this data, it is possible to accurately capture and analyze the corrosion rate's dependence on stress, which is a key aspect of the current work. This inclusion adds a novel and crucial dimension to the work, providing valuable insights into the relationship between stress and corrosion.

The mesh employed in this model is a free triangular mesh, comprising two distinct sizes. An extremely fine mesh resolution is utilized specifically for the alpha phase, which represents the corrosion defect. This finer mesh resolution ensures enhanced accuracy of the results in this critical region. On the other hand, a fine mesh resolution is employed for the rest of the geometry, offering a suitable balance between computational efficiency and result quality.

### 2.2 Mechanical model

FEM serves as a robust tool for assessing the structural integrity of pipelines containing defects arising from mechanical and electrochemical factors. The application of FEA allows for an effective modeling of the failure process in such pipelines. For pipelines affected by corrosion defects, crucial factors influencing the remaining strength include the dimensions of the corrosion defect (e.g. length, width, and depth), as well as the number of defects. In this study, the focus lies on investigating the influence of corrosion defect depth on burst pressure prediction, while keeping the length and width of the defect constant, utilizing Abaqus. Here propagation of cracks through the corrosion defect will be examined using the extended finite element method (XFEM) similar to the approach outlined in Ref. [19]. The material properties for the pipe grade API X100 considered in this study are listed in Table 1.

**Table 1:** X100 steel mechanical properties [20].

API Grade	Young's Modulus *E*	Poisson's Ratio ν	Yield Strength *S*_*y*_	Tensile Strength *S*_*ut*_	Elongation	Fracture Toughness *K*_*Ic*_
	GPa	–	MPa	MPa	%	$\text{MPa}\sqrt{m}$
X100	210	0.33	717	786	19	75.5

It is important to note that this work does not account for the impact of fluid flow within the pipeline during operation or the thermal stresses resulting from the transportation of high-temperature fluids. The pipeline is assumed to be solely influenced by the internal pressure, neglecting the effects of fluid flow and thermal stresses [21]. However, the working load and internal pressure are considered as significant factors influencing the pipeline behavior in this study.

#### 2.2.1 Model geometry

The geometry employed in this study consists of an axisymmetric cross-section of a pipeline. The dimensions of the model are defined as follows: a length *L* = 100 mm, thickness *t* = 10 mm, and inner diameter ID = 300 mm. In order to simulate real-world conditions, an elliptical defect is incorporated at the center of the pipeline's external surface.

The selection of different defect dimensions in the analysis are tabulated in Table 2 and can be justified by considering the realistic conditions observed in the corrosion model, as discussed in the preceding section. In this study, the width of the defect is kept constant throughout all three trials to maintain consistency and isolate the influence of the defect depth on the system's behavior. The defect depth values in Table 2 are chosen based on values found in the literature [12,13,22,23] and reflect the real-world conditions observed in the corrosion process.

**Table 2:** Defect dimensions.

Trail	Time (s)	Defect depth (mm)	Defect length (mm)
1	0	0.5 mm	2.0 mm
2	5	0.8 mm	2.0 mm
3	10	1.5 mm	2.0 mm

#### 2.2.2 Material parameters

The mechanical response of the pipe is characterized as elasto-plastic with isotropic hardening, with material properties given by Table 1, which also provides a summary of the input material properties utilized in the XFEM model, adapted from Ref. [20] for API X100 pipe grade. The criterion for damage initiation in this model is based on the maximum principal stress, while the damage evolution is determined from the fracture energy $G_{Ic}$, which is calculated using the following relation:

$$G_{Ic}=\frac{{K_{Ic}}^{2}\left(1-\nu^{2}\right)}{E}$$

where the fracture toughness *K*_*Ic*_ value is obtained from Table 1 specifically for the X100 steel pipeline with a thickness *t* = 10 mm.

#### 2.2.3 Boundary conditions and loads

Transportation pipelines are characterized by their extensive length, and in cases where corrosion is present, its impact is localized to the region surrounding the corrosion defect. Consequently, the model focuses on simulating only the corroded portion of the pipeline, considering it as a representative segment. To establish an accurate representation, a symmetry condition is applied to both ends of the pipeline. As a result, the bottom and upper ends are fixed, restricting any longitudinal movement in the simulation. This approach ensures that the simulated model aligns with the actual behavior of the pipeline under study, taking into account the boundary conditions and constraints imposed by the fixed ends.

The loading conditions experienced by pipelines are inherently complex, encompassing factors such as pipeline weight, concentrated loads, and various other loads. Among these, internal pressure plays a predominant role in influencing stress concentrations within pipelines. Therefore, internal pressure is considered as the sole mechanical load applied to the system. Initially, a constant internal pressure is imposed on the pipeline model, with a magnitude of 120 MPa.

The accuracy of the results are highly dependent on the mesh employed throughout the analysis, where a finer mesh give rise to higher accuracy. However, utilizing a refined mesh necessitates more powerful computational resources and longer computation times. Consequently, the selection of mesh divisions should be carefully tailored to the model's size and the desired level of calculation accuracy.

In the case of a pipeline with a corrosion defect, a larger grid size is chosen for the unaffected body region to optimize computation time, while finer mesh sizes are implemented in the corrosion-affected areas to ensure accuracy and convergence of the calculations. This is due to the significant variation in stress and strain gradients observed in corrosion-affected regions. Here a structured mesh is employed with a seed size of 1 mm for the overall model body, while a finer mesh with a seed size of 0.1 mm is utilized at the site of the corrosion defect. Given that failure occurs primarily within the corroded region, and crack propagation originates from the center of the corrosion defect, a larger number of elements are concentrated in this specific area.

## 3 Results and discussion

### 3.1 COMSOL multiphysics model

**Fig. 3:**
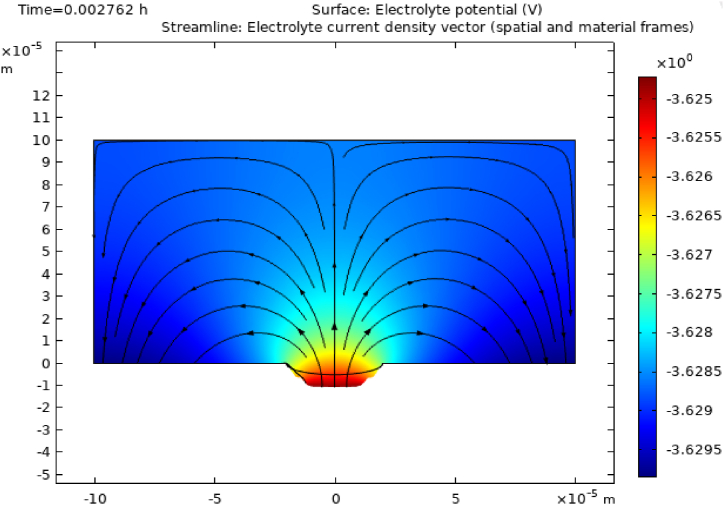
Surface electrolyte potential at time 0.002762 h.

The electrolyte potential distribution in conjunction with an elliptical corrosion defect at time 0.002762 h is shown in Fig. 3. This surface plot offers valuable insights into the electrochemical behavior and localized corrosion dynamics within the system. It is evident from these results that the electrolyte potential exhibits a distinct spatial variation, with particularly high values observed at the center of the corrosion defect. Moving away from the corrosion defect center, the potential gradually decreases. This observation aligns with the findings from Ref. [3] where an increase in corrosion defect depth leads to localized stress concentration at the center of the defect. Both studies demonstrate the protective capabilities of the beta phase, which remains relatively intact due to its electrochemical protection mechanisms and the formation of a protective oxide layer on its surface.

The protection of the beta phase arises from its inherent passivation behavior, wherein a protective oxide layer forms on its surface. This oxide layer acts as a barrier, impeding the electrochemical reactions responsible for the dissolution of the beta phase. Furthermore, the presence of surface layers on the beta phase may occur, contingent upon the ion concentration in the electrolyte and the respective surface-electrolyte potentials. The deposition of surface layers on the beta phase further enhances its protection by impeding the diffusion of corrosive species and reducing electrochemical activity at the interface. These observations offer valuable insights into the localized corrosion mechanisms operating within the system. Understanding the distinct behaviors of the alpha and beta phases aids in understanding the corrosion resistance and the protective capabilities of different material phases. This knowledge can inform the development of targeted corrosion mitigation strategies, including the optimization of material composition and surface treatments, to enhance overall performance and durability in corrosive environments. Further investigations are warranted to delve deeper into the underlying mechanisms driving the observed electrochemical phenomena and to refine strategies for effective corrosion management in this particular system.

**Fig. 4:**
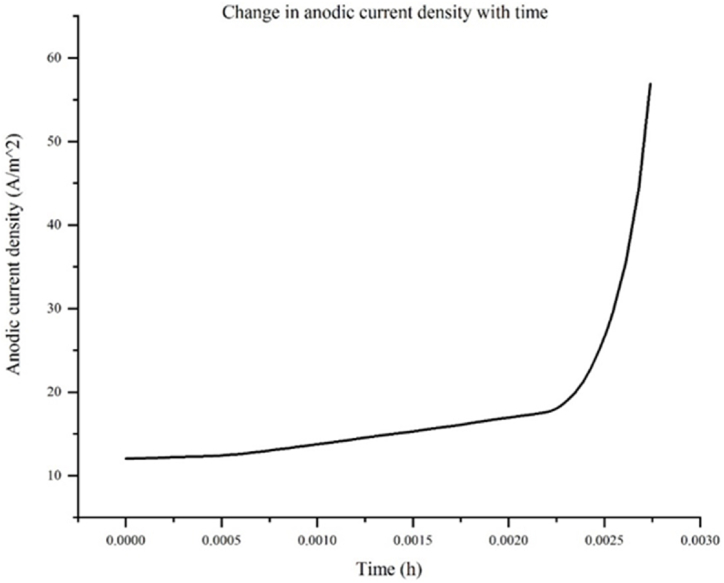
Change in anodic current density with time.

Fig. 4 presents the temporal evolution of the average anodic current density along the corrosion defect, e.g. the corrosion growth rate. The observed results unveil a distinctive trend wherein the average anodic current density exhibits a gradual increase in its magnitude initially, followed by a subsequent rapid escalation. This behavior can be attributed to several underlying factors related to the composition and morphology of the corroded surface. The initial gradual increase in the average anodic current density corresponds to regions where the fraction of the surface beta phase is relatively low. This suggests that the beta phase, characterized by its nobler nature, exhibits a higher resistance to anodic dissolution compared to the alpha phase. Consequently, areas with a lower proportion of the beta phase experience a relatively higher anodic current density as the less noble alpha phase undergoes dissolution at a faster rate.

The subsequent rapid escalation of the average anodic current density can be justified by two primary factors: the higher cathode to anode area ratio at the electrode surface and the pronounced stress concentration present at the corrosion defect. The higher cathode to anode area ratio arises due to the irregularities and uneven distribution of corrosion activity along the defect, resulting in local variations in the current density. Consequently, regions with a larger cathode area, characterized by the presence of the noble beta phase, contribute to the higher average anodic current density.

Additionally, the stress concentration present at the corrosion defect significantly diminishes the corrosion resistance of the steel. This elevated stress concentration acts as a localized site for increased electrochemical reactions, thereby promoting accelerated corrosion. The compromised corrosion resistance exacerbates the anodic dissolution rate, leading to a substantial increase in the average anodic current density.

Furthermore, the enhanced anodic dissolution and subsequent increase in the average anodic current density contribute to a rise in the corrosion potential. The heightened corrosion potential further amplifies the corrosion rate, creating a positive feedback loop that sustains and accelerates the corrosion process. Thus, the defect shapes and sizes obtained from the results of the electrochemical model, e.g. COMSOL Multiphysics model, as listed in Table 2 are imported into the mechanical model (e.g. XFEM model) at various time increments in order to predict crack initiation and propagation from these external corrosion defects when an internal pressure is present.

### 3.2 XFEM model

**Fig. 5:**
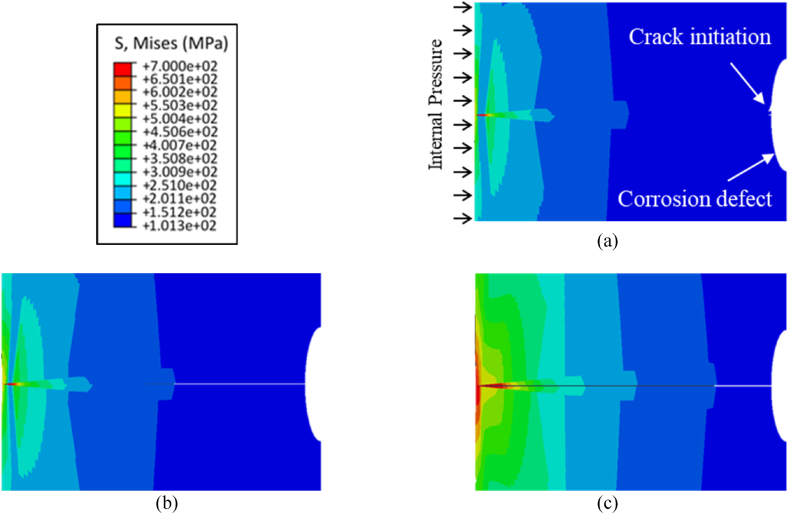
Results from initial defect depth of 0.5 mm model (a) initiation of crack at an internal pressure of 68.4 MPa (b) propagation of crack through the pipe thickness (c) crack reaching the bottom of the pipeline surface.

**Fig. 6:**
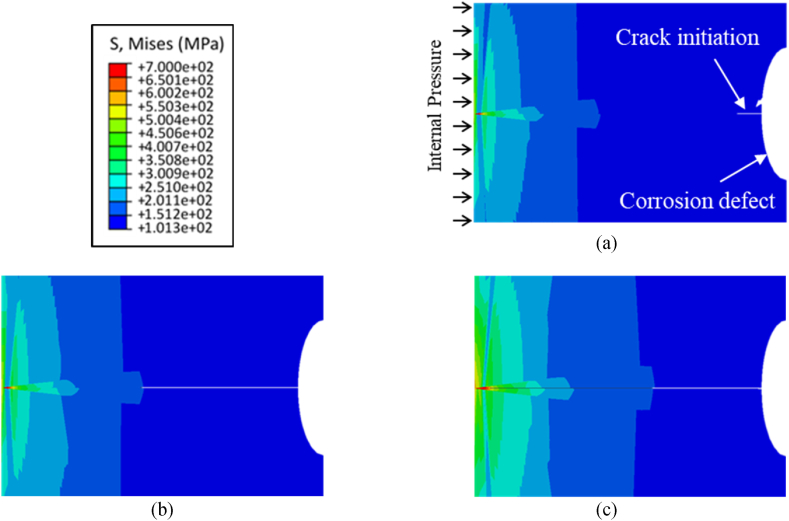
Results from defect depth of 0.8 mm model (a) initiation of crack at an internal pressure of 52.0 MPa (b) propagation of crack through the pipe thickness (c) crack reaching the bottom of the pipeline surface.

**Fig. 7:**
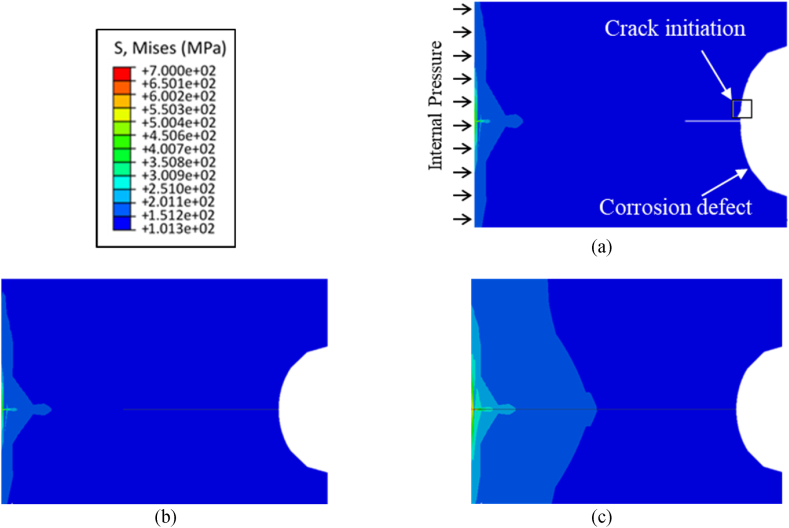
Results from defect depth of 1.5 mm model (a) initiation of crack at an internal pressure of 21.8 MPa (b) propagation of crack through the pipe thickness (c) crack reaching the bottom of the pipeline surface.

As mentioned in the previous section, the initial external defect shapes and sizes in the XFEM model are based on the results from the electrochemical model, and the objective of the mechanical model is to predict initiation and propagation of a crack from these external corrosion defects when the pipe is subjected to internal pressure. The crack initiation and propagation behavior of pipelines containing corrosion defects is illustrated in Fig. 5, pertaining to corrosion defect depth of 0.5 mm. The crack initiation is observed at an internal pressure of 64.8 MPa for the initial defect depth of 0.5 mm, as depicted in Fig. 5 (a), coinciding with the maximum stress at the internal surface of the pipeline reaching 700 MPa. Subsequently, with increasing pressure, the crack initiates and propagates through additional elements within the pipeline. The extent of crack propagation is illustrated in Fig. 5 (b), while Fig. 5 (c) displays the point at which the crack fully traverses the pipeline's thickness. The crack initiation starts at 52.0 MPa for a defect depth of 0.8 mm as shown in Fig. 6 (a), and it propagates as shown in Fig. 6 (b) until it reaches the bottom of the pipe thickness as indicated in Fig. 6(c). For a defect depth of 1.5 mm, the crack initiation, crack propagation and failure of the pipe are shown in Fig. 7. Comparative analysis of the results presented in Fig. 5, Fig. 6, Fig. 7 indicates that the failure pressure decreases as the defect depth increases, as expected. It is noteworthy that the material in all three models exhibits some degree of plasticity before the crack propagates through the pipeline's thickness, indicative of a more ductile response. This behavior can be attributed to the elastic-plastic properties of the material, wherein limited plastic deformation occurs prior to crack propagation.

**Fig. 8:**
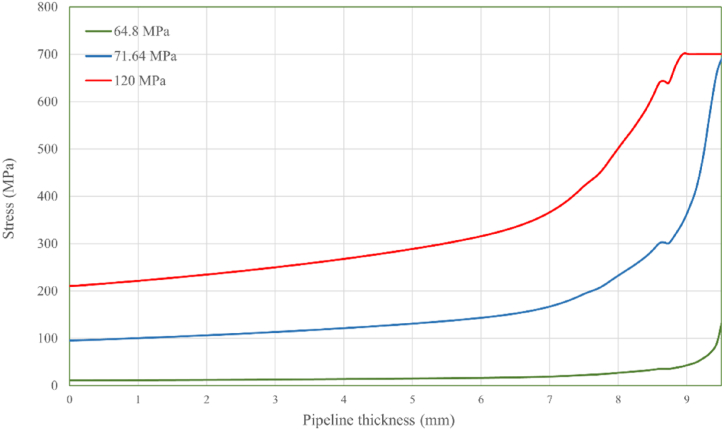
von Mises stress vs. crack path for defect depth of 0.5 mm at 3 internal pressure levels.

**Fig. 9:**
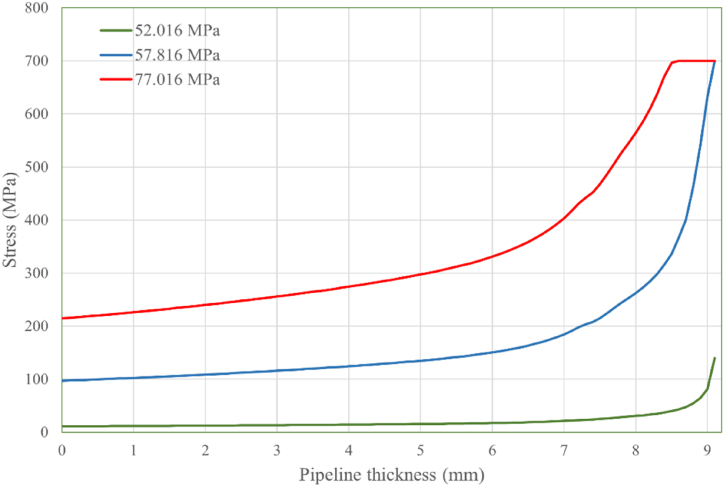
von Mises stress vs. crack path for defect depth of 0.8 mm at 3 internal pressure levels.

**Fig. 10:**
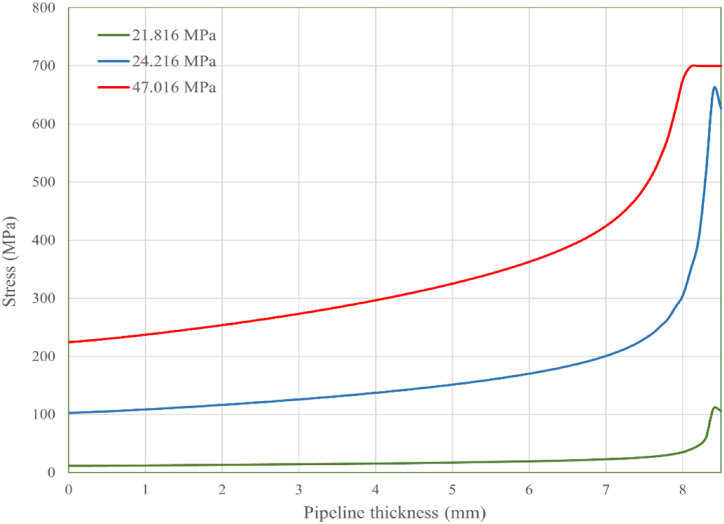
von Mises stress vs. crack path for defect depth of 1.5 mm at 3 internal pressure levels.

**Fig. 11:**
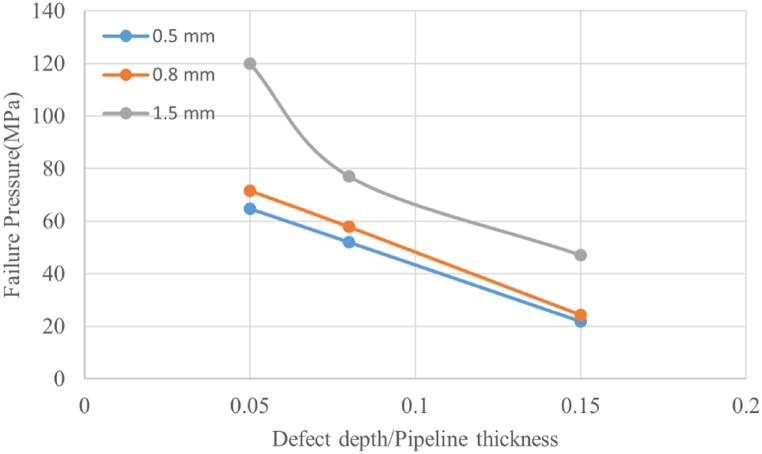
Failure pressure for different defect depths with varying defect depth/pipeline thickness ratio.

The von Mises stress distribution along the crack path, corresponding to defect depths of 0.5 mm, 0.8 mm, and 1.5 mm at various internal pressure levels, is displayed in Fig. 8, Fig. 9, Fig. 10, respectively. The results for all three defect depths demonstrate an increase in intensity in von Mises stress as the pipeline's thickness decreases. This disparity can be attributed to the progressive accumulation of stress concentration at the internal surface of the pipeline over time due to the applied internal loading conditions. The stress concentration acts as a driving force for crack growth and propagation along the designated crack path. In Fig. 11 the predicted failure pressure against the defect depth/pipeline thickness ration is presented, revealing that the failure pressure decreases with increasing defect depth and decreasing pipeline thickness. This observation is expected since an increase in corrosion defect depth corresponds to a reduction in pipeline thickness, rendering the pipeline more susceptible to crack propagation and, consequently, failure.

## 4 Conclusions

In this study, we employ two numerical models to investigate the corrosion kinetics and crack propagation behavior of buried X100 steel pipelines with pre-existing corrosion defects. The first model, implemented using COMSOL Multiphysics, provided valuable insights into the anodic current density and corrosion dynamics over time. The results indicated a consistent increase in the anodic current density with the passage of time, highlighting the progressive nature of corrosion.

To further analyze the crack initiation and propagation phenomenon, a mechanical model based on XFEM is developed in Abaqus. Three different defect depths, namely 0.5 mm, 0.8 mm, and 1.5 mm, were considered in this model. The findings obtained from the XFEM simulations revealed a strong correlation between the corrosion defect depth and the crack initiation and propagation behavior of the pipeline. Specifically, it was observed that as the defect depth increased, both the crack initiation and propagation occurred at a faster rate. This suggests that deeper corrosion defects are more prone to rapid crack development, posing a significant threat to the structural integrity of the pipeline. Furthermore, the analysis of von Mises stress distribution along the crack path provided insights into the mechanical response of the defected pipeline. The results demonstrated that the rate of stress increase was considerably higher for deeper corrosion defects compared to shallower ones. This implies that pipelines with deeper defects experience greater stress concentrations, which accelerate crack growth and propagation.

These findings underscore the critical influence of corrosion defect depth on the crack initiation, propagation, and mechanical behavior of the pipeline. It emphasizes the importance of proactive corrosion management strategies, including regular inspection and maintenance, to identify and address corrosion defects at an early stage. The findings of this research make a valuable contribution to the comprehension of failure mechanisms resulting from corrosion and offer insights that can facilitate the enhancement of corrosion mitigation strategies and pipeline integrity management. However, it is important to acknowledge that additional investigations are necessary to explore other factors and parameters that influence corrosion kinetics and the behavior of crack propagation in real-world pipeline systems.

## Data availability

The data supporting the findings in this study are available within this paper, and any other raw data supporting the findings of this paper are available from the author upon reasonable request. We are committed to transparency and reproducibility, and we encourage fellow researchers to reach out if they require additional information or datasets related to this study.

## CRediT authorship contribution statement

**Ghadeer Mubarak:** Investigation, Methodology, Validation, Writing – original draft, Writing – review & editing. **Ibrahim Gadala:** Methodology, Writing – review & editing. **Imad Barsoum:** Methodology, Supervision, Writing – review & editing. **Akram AlFantazi:** Supervision, Writing – review & editing.

## Declaration of competing interest

The authors declare that they have no known competing financial interests or personal relationships that could have appeared to influence the work reported in this paper.
